# Donepezil ameliorates Aβ pathology but not tau pathology in 5xFAD mice

**DOI:** 10.1186/s13041-022-00948-1

**Published:** 2022-07-18

**Authors:** Hee-Jeong Choi, Jin-Hee Park, Yoo Joo Jeong, Jeong-Woo Hwang, Soojung Lee, Heeyong Lee, Eunyoung Seol, Ik-whi Kim, Byung-Yoon Cha, Jinsoo Seo, Minho Moon, Hyang-Sook Hoe

**Affiliations:** 1 Department of Neural Development and Disease, Korea Brain Research Institute (KBRI), 61, Cheomdan-ro, Dong-gu, 41068 Daegu, Republic of Korea; 2 Department of Brain Sciences, Daegu Gyeongbuk Institute of Science & Technology, 42988 Daegu, Korea; 3 G2GBIO, Inc., Science Park#411, 1646 Yuseond-daero, Yuseong-gu, 34054 Daejeon, Korea; 4 PharmacoRex Co., Ltd., 20 Techno 1-ro, Yuseong-gu, 34016 Daejeon, Korea; 5 Department of Biochemistry, College of Medicine, Konyang University, 35365 Daejeon, Korea

**Keywords:** Alzheimer’s disease, Tau, Tau kinase, Amyloid beta, 5xFAD mice, Donepezil

## Abstract

The cholinesterase inhibitor donepezil is used to improve Aβ pathology and cognitive function in patients with Alzheimer’s disease (AD). However, the impact of donepezil on tau pathology is unclear. Thus, we examined the effects of donepezil on Aβ and tau pathology in 5xFAD mice (a model of AD) in this study. We found that intraperitoneal injection of donepezil (1 mg/kg, i.p.) exhibited significant reductions in Aβ plaque number in the cortex and hippocampal DG region. In addition, donepezil treatment (1 mg/kg, i.p.) reduced Aβ-mediated microglial and, to a lesser extent, astrocytic activation in 5xFAD mice. However, neither intraperitoneal/oral injection of donepezil nor oral injection of rivastigmine altered tau phosphorylation at Thr212/Ser214 (AT100), Thr396, and Thr231 in 5xFAD mice. Surprisingly, we observed that intraperitoneal/oral injection of donepezil treatment significantly increased tau phosphorylation at Thr212 in 5xFAD mice. Taken together, these data suggest that intraperitoneal injection of donepezil suppresses Aβ pathology but not tau pathology in 5xFAD mice.

**Supplementary Information:**

The online version contains supplementary material available at 10.1186/s13041-022-00948-1.

Alzheimer’s disease (AD) is a neurodegenerative disease that reduces neurocognitive ability [1]. The two neuropathological symptoms of AD are amyloid-β (Aβ) and tau deposition, which are consequently major targets for the development of AD treatments [2]. Unfortunately, most drugs targeting Aβ or tau (single target) have failed in clinical studies [3, 4]. Currently used AD treatments include the acetylcholinesterase inhibitor donepezil, which regulates learning/memory, neuroinflammation, and Aβ pathology in AD patients [5–7], but the impact of donepezil on tau phosphorylation has received limited attention.

To address this gap, we examined the effects of donepezil on Aβ pathology in 5xFAD mice, a model of AD. For these experiments, 5xFAD mice were intraperitoneally (i.p.) injected with vehicle or donepezil (1 mg/kg, i.p.) daily for 2 weeks, followed by immunofluorescence staining with an anti-6E10 antibody. We found that 5xFAD mice treated with donepezil exhibited significant decreases in Aβ plaque number (Fig. 1A–E). In addition, Aβ-mediated microglial activation and, to a lesser extent, Aβ-induced astrocyte activation were significantly reduced in donepezil-treated 5xFAD mice (Fig. 1 A–D, F–G). These data suggest that intraperitoneal injection of donepezil affects Aβ plaque number and Αβ-stimulated glial activation in 5xFAD mice.

**Fig. 1:**
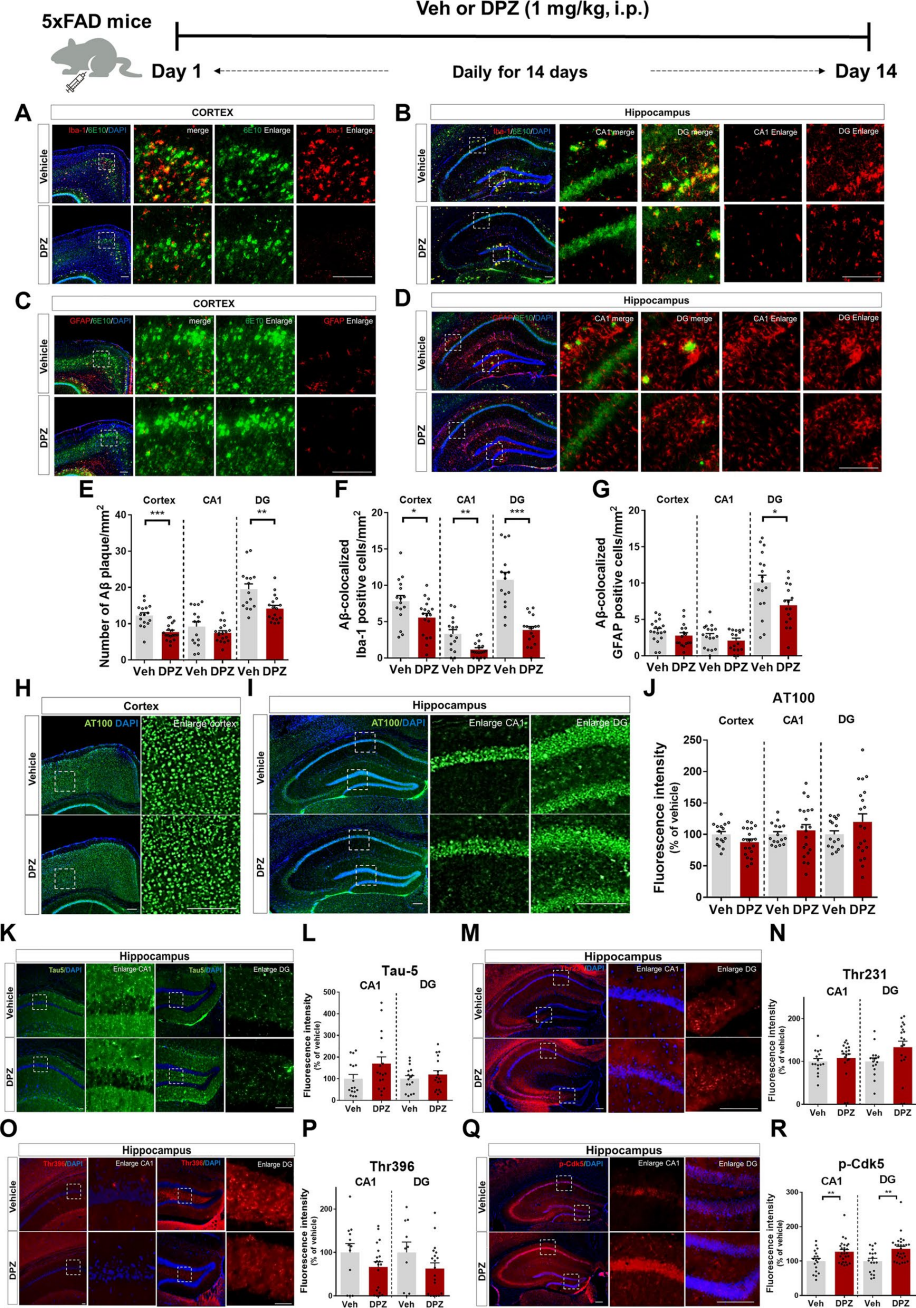
Intraperitoneal injection of donepezil downregulates Aβ pathology but not tau pathology in 5xFAD mice. 5xFAD mice were injected with vehicle (veh) or donepezil (1 mg/kg, i.p.) daily for 2 weeks, and immunofluorescence staining was conducted. **A**–**G** Representative images of immunohistochemical staining with Aβ pathology-related antibodies (veh, n = 4; DPZ, n = 5/group). **H**–**P** Representative images of immunohistochemical staining with tau pathology-related antibodies (veh, n = 4; DPZ, n = 5/group). **Q**–**R** Representative images of immunohistochemical staining with tau kinase p-Cdk5 antibody (veh, n = 4; DPZ, n = 5). Scale bar = 100 or 200 μm. *p < 0.05; **p < 0.01; ***p < 0.001

Next, we investigated the impact of oral injection of donepezil on Aβ pathology. For this experiment, 5xFAD mice were orally injected with vehicle or donepezil (3 mg/kg, p.o.) daily for 2 weeks. In addition, wild-type mice were orally injected with vehicle daily for 2 weeks. We found that oral administration of donepezil did not significantly alter Aβ plaque number and Aβ-mediated microglial activation in 5xFAD mice (Additional file 1: Fig. S1). However, oral administration of donepezil significantly reduced Aβ-mediated astrocyte activation in the CA1 region (Additional file 1: Fig. S1). Based on our observations and the literature, it is possible that long-term oral injection of donepezil (daily for 3 or 5 months) would affect Aβ pathology in 5xFAD mice. A future study will address this possibility.

We then examined the effects of donepezil on tau pathology in 5xFAD mice. Consistent with previous findings, tau phosphorylation was higher in vehicle-treated 5xFAD mice than in vehicle-treated wild-type mice [8]. Moreover, neither intraperitoneal nor oral injection of donepezil altered tau phosphorylation at Thr212/Ser214, Thr231, or Thr396 or total tau levels compared with vehicle-treated 5xFAD mice (Fig. 1 H-P, Additional file 1: Figs. S2, S3). Surprisingly, we found that tau phosphorylation at Thr212 was significantly enhanced by donepezil treatment Additional file 1: Figs. S2, S3). These data indicate that donepezil has a negative or no impact on tau phosphorylation in 5xFAD mice, depending on the phosphorylation site. We subsequently explored the molecular mechanism by which donepezil affects tau pathology and found that intraperitoneal administration of donepezil significantly upregulated tau kinase p-Cdk5 levels in 5x FAD mice (Fig. 1Q-R).

To test whether other acetylcholinesterase inhibitors can modulate tau phosphorylation in a mouse model of AD, 5xFAD mice were orally injected with rivastigmine (2 mg/kg, p.o.) or vehicle daily for 2 weeks. We found that oral administration of rivastigmine did not alter tau phosphorylation at Thr212/Ser214, Thr396, or Thr212 in 5xFAD mice (Additional file 1: Fig. S4).

In summary, intraperitoneal administration of donepezil (1 mg/kg, daily for 2 weeks, i.p.) significantly reduced Aβ plaque number and Aβ-stimulated glial activation in 5xFAD mice. However, neither intraperitoneal nor oral injection of donepezil nor oral injection of rivastigmine positively affected tau phosphorylation in 5xFAD mice. Taken together, these data suggest that intraperitoneal injection of donepezil downregulates Aβ pathology and that neither intraperitoneal nor oral administration of donepezil alters tau phosphorylation in 5xFAD mice.

As mentioned above, we and others have found that donepezil improves learning, memory, Aβ pathology, and neuroinflammation in mouse models of AD [5, 7, 9], but studies of the effects of donepezil on tau pathology are scarce. Interestingly, a previous study demonstrated that injection of tau-overexpressing PS19 mice with donepezil (8 month injection period) significantly reduced tau phosphorylation at Ser202/Thr205 [10]. Another study found that donepezil treatment (1.3 mg/kg, intragastrically once daily) did not alter tau phosphorylation at Ser202, Ser396, or Ser416 [11]. These conflicting findings on the effects of donepezil on tau pathology may be due to differences in AD mouse models (tau-overexpressing PS19 mice vs. APP/PS1 and APP/PS1/Tau Tg mice), donepezil administration methods/durations, and donepezil dosages. In the present study, we assessed tau pathology in 3- to 4-month-old Aβ-overexpressing 5xFAD mice or wild-type mice that were injected with donepezil or vehicle daily for 2 weeks. The wild-type mice expressed basal levels of tau phosphorylation, consistent with previous findings [11]. In 5xFAD mice, most sites of tau phosphorylation (Thr212/Ser214, Thr231, and Thr396) were unaffected by donepezil or rivastigmine treatment (Fig. 1, Additional file 1: Figs. S3, S4). Based on the literature and our findings, it is possible that long-term injection or lower/higher doses (e.g., 0.7 or 1.3 mg/kg) of donepezil or rivastigmine might have different effects on tau pathology in 5xFAD mice, which will be examined in future work.

In addition, intraperitoneal injection of donepezil significantly increased tau phosphorylation at Thr212 and tau kinase p-Cdk5 levels (Fig. 1Q-R, Additional file 1:  Fig. S2). It is possible that the increase in tau phosphorylation at Thr212 was due to activation of the tau kinase p-Cdk5. Another possibility is that donepezil modulated another tau kinase (e.g., DYRK1A or p-GSK3β) to directly and/or indirectly affect tau phosphorylation. We will evaluate the effects of donepezil on the activation of other tau kinases and tau pathology in young and old 5xFAD mice (3 and 8 months old) in a future study.

In conclusion, intraperitoneal injection of donepezil daily for 2 weeks suppressed Aβ plaque levels and Aβ-stimulated glial activation in 5xFAD mice. Oral or intraperitoneal injection of donepezil or oral injection of rivastigmine daily for 2 weeks did not positively affect tau phosphorylation in 5xFAD mice. Taken together, our findings suggest that intraperitoneal administration of donepezil inhibits Aβ pathology and that neither intraperitoneal/oral administration of donepezil nor oral administration of rivastigmine regulates tau pathology in 5xFAD mice.

## Supplementary Information

**Additional file 1:** **Fig. S1**. Oral injection of 3 mg/kg donepezil does not affect Aβ plaque number in 5xFAD mice. **Fig. S2**. Intraperitoneal administration of donepezil does not alter total tau levels but significantly increases tau phosphorylation at Thr212 in 5xFAD mice. **Fig. S3**. Oral injection of 3 mg/kg donepezil does not alter tau phosphorylation at Thr212/Ser214 and Thr396 but significantly increases tau phosphorylation at Thr212 in 5xFAD mice. **Fig. S4.** Oral injection of rivastigmine does not affect tau phosphorylation in 5xFAD mice. Additional Materials and Methods section.

## Data Availability

All data generated and/or analyzed during this study are included in this published article and its Additional information. Materials and methods are presented in the Additional information.

## Abbreviations

Aβ: Amyloidβ
AD: Alzheimer’s disease
Cdk-5: Cyclin-dependent kinase 5
DG: Dentate gyrus
GSK-3β: Glycogen synthase kinase-3β
