# Supplementary material for: Donepezil ameliorates Aβ pathology but not tau pathology in 5xFAD mice
Source: Mol Brain. 2022 Jul 18;15:63. doi: 10.1186/s13041-022-00948-1 (PMC9290238; doi:10.1186/s13041-022-00948-1)
Supplement: Supplementary file 1 — Additional file 1: Fig. S1. Oral injection of 3 mg/kg donepezil does not affect Aβ plaque number in 5xFAD mice. Fig. S2. Intraperitoneal administration of donepezil does not alter total tau levels but significantly increases tau phosphorylation at Thr212 in 5xFAD mice. Fig. S3. Oral injection of 3 mg/kg donepezil does not alter tau phosphorylation at Thr212/Ser214 and Thr396 but significantly increases tau phosphorylation at Thr212 in 5xFAD mice. Fig. S4. Oral injection of rivastigmine does not affect tau phosphorylation in 5xFAD mice. Additional Materials and Methods section. [file 13041_2022_948_MOESM1_ESM.docx]

**Donepezil ameliorates Aβ pathology but not tau pathology in 5xFAD mice**

Hee-Jeong Choi^1,6^, Jin-Hee Park^1,2,6^, Yoo Joo Jeong^1^, Jeong-Woo Hwang^1^, Soojung Lee^3^, Heeyong Lee^3^, Eunyoung Seol^3^, Ik-whi Kim^4^, Byung-Yoon Cha^4^, Jinsoo Seo^2^, Minho Moon^5,^*, Hyang-Sook Hoe^1,2,*^

^1^Department of Neural Development and Disease, Korea Brain Research Institute (KBRI), 61, Cheomdan-ro, Daegu, Republic of Korea; ^2^Department of Brain Sciences, Daegu Gyeongbuk Institute of Science & Technology, Daegu 42988, Korea; ^3^G2GBIO, Inc., Science Park#411, 1646 Yuseond-daero, Yuseong-gu, Daejeon, Korea, 34054; ^4^PharmacoRex Co., Ltd., 20 Techno 1-ro, Yuseong-gu, Daejeon, Korea, 34016; ^5^Department of Biochemistry, College of Medicine, Konyang University, Daejeon, Korea, 35365; ^6^These authors contributed equally to this work.

^*^Corresponding author

Hyang-Sook Hoe, Ph.D., Department of Neural Development and Disease, Korea Brain Research Institute (KBRI), 61, Cheomdan-ro, Dong-gu, Daegu, Korea 41068

E-mail: [sookhoe72@kbri.re.kr](mailto:sookhoe72@kbri.re.kr)

Minho Moon, Ph.D., Department of Biochemistry, College of Medicine, Konyang University, Daejeon, Korea, 35365

E-mail: hominmoon@konyang.ac.kr

**Additional file 1: Fig. S1**. Oral injection of 3 mg/kg donepezil does not affect Aβ plaque number in 5xFAD mice. Three-month-old male wild-type mice or 5xFAD mice were orally injected with vehicle (veh) or donepezil (3 mg/kg, p.o.) daily for 14 days and subsequently sacrificed for immunofluorescence staining. (A-D) Representative images of immunohistochemical staining with anti-6E10 and anti-Iba-1 antibodies or anti-6E10 and anti-GFAP antibodies. (E-G) Quantification of data from A-D (WT-Veh, n = 5; 5xFAD-Veh, n = 5; 5xFAD-DPZ, n = 4). Scale bar = 200 μm. ***p* < 0.01; ****p* < 0.001

**Additional file 1: Fig. S2**. Intraperitoneal administration of donepezil does not alter total tau levels but significantly increases tau phosphorylation at Thr212 in 5xFAD mice. Three-month-old male 5xFAD mice were intraperitoneally injected with vehicle (veh) or donepezil (1 mg/kg, i.p.) daily for 14 days and subsequently sacrificed for immunofluorescence staining. (A, B) Representative images of immunohistochemical staining with an antibody against Tau-5 or against tau phosphorylated at Thr212. (C, D) Quantification of data from A and B (Veh, n = 4; DPZ, n = 5/group). Scale bar = 200 μm. ****p* < 0.001

**Additional file 1: Fig. S3**. Oral injection of 3 mg/kg donepezil does not alter tau phosphorylation at Thr212/Ser214 and Thr396 but significantly increases tau phosphorylation at Thr212 in 5xFAD mice. Three-month-old male wild-type mice or 5xFAD mice were orally injected with vehicle (veh) or donepezil (3 mg/kg, p.o.) daily for 14 days and subsequently sacrificed for immunofluorescence staining. (A-C) Representative images of immunohistochemical staining with anti-AT100, anti-Tau^Thr396^, and anti-Tau^Thr212^ antibodies. (D-F) Quantification of data from A-C (WT-Veh, n = 5; 5xFAD-Veh, n = 5; 5xFAD-DPZ, n = 4)**.** Scale bar = 200 μm. ***p* < 0.01

Additional file 1: Fig. S4. Oral injection of rivastigmine does not affect tau phosphorylation in 5xFAD mice. Three-month-old male 5xFAD mice were orally injected with vehicle (veh) or rivastigmine (2 mg/kg, p.o.) daily for 14 days and subsequently sacrificed for immunofluorescence staining. (A-C) Representative images of immunohistochemical staining with anti-AT100, anti-Tau^Thr396^, and anti-Tau^Thr212^ antibodies. (D-F) Quantification of data from A-C. (Veh, n = 4; Riva, n = 5). Scale bar = 200 μm.

Materials and Methods

**Drugs**

For *in vivo* experiments, 5xFAD mice were intraperitoneally injected with vehicle or donepezil (1 mg/kg, i.p.; Neuland Laboratories Limited, Cat. no. 120014-70-3) daily for 2 weeks. The effects of oral injection of donepezil on AD pathology were determined using three groups of mice: wild-type mice injected with saline; 5xFAD mice injected with saline; 5xFAD mice injected with donepezil (3 mg/kg, p.o.). In addition, to examine the effects of rivastigmine on tau pathology, 5xFAD were orally injected with PBS or rivastigmine (2 mg/kg, p.o.). Each treatment was administered daily for 2 weeks. After 2 weeks, brain tissues from sacrificed mice were subjected to immunofluorescence staining.

**Animals**

All animal experimental protocols and guidelines were approved by the Korea Brain Research Institute Animal Care and Use Committee (IACUC-21-00021). Mice were housed at 22±2°C and 50±5% humidity in a pathogen-free facility with a 12-h photoperiod and free access to chow diet and water.

**Wild-type mice and 5xFAD mice (AD mouse model)**

C57BL6 mice (wild-type, male, 3 months old, weight 20-25 g) were used in in vivo experiments. In addition, 5xFAD mice (Stock No. 34848-JAX, Jackson Laboratory, Bar Harbor, ME, USA) were used as a model of AD in this study, which is overexpressing human APP and PS1 with five familial AD mutations (B6.Cg-Tg (APPSwFlLon, PSEN1*M146L*L286V)6799Vas/Mmjax).

**Immunofluorescence staining**

Brain tissues were fixed, and a cryostat microtome (Leica CM1850, Wetzlar, Germany) was used to obtain sections with a thickness of 30 μm. The sections were blocked with 4% normal goat serum (Vector Laboratories, Burlingame, CA, USA) for 2 h at room temperature and subsequently immunostained with primary antibodies (anti-Iba-1, anti-GFAP, anti-6E10, anti-AT100, anti-Tau-5, anti-Tau^Thr231^ anti-Tau^Thr396^, anti-Tau^Thr212^, or anti-p-Cdk5) at 4°C overnight. Two or three days later, the tissues were then washed 3 times with PBST buffer and incubated with Alexa 555- or Alexa 488-conjugated secondary antibodies for 2 h at room temperature. Two hours later, the tissues were then washed 3 times with PBST/PBST with DAPI solution/PBS buffer and mounted in antifade mounting medium (Vector Laboratories, Burlingame, CA, USA). Fluorescence microscopy (DMi8, Leica Microsystems, Wetzlar, Germany) images of immunostained tissue mounted on glass slides using Vectashield mounting solution with DAPI (Vector Labs, Burlingame, CA) were analyzed using ImageJ (US National Institutes of Health, Bethesda, MD, USA).

**Statistical analysis**

Results are presented as means ± SED (**p < 0.05*, ***p < 0.01*, and ****p < 0.001*). The software Prism 7 (GraphPad Software, USA) was used to analyze the significance of differences between groups. Pairwise comparisons were performed using unpaired two-tailed t-tests with Welch’s correction, and multiple comparisons were conducted using one-way analysis of variance (ANOVA) with Tukey’s or Dunnett’s multiple comparisons test.
